# In vitro culture of the zoonotic nematode *Anisakis pegreffii* (Nematoda, Anisakidae)

**DOI:** 10.1186/s13071-022-05629-5

**Published:** 2023-02-02

**Authors:** Ivona Mladineo, Artemis Charouli, Filip Jelić, Anand Chakroborty, Jerko Hrabar

**Affiliations:** 1 Laboratory of Functional Helminthology, Institute of Parasitology, Biology Centre Czech Academy of Sciences, Ceske Budejovice, Czechia; 2 Cross-Border Study of Biological Chemistry, Faculty of Science, University of South Bohemia, Ceske Budejovice, Czechia; 3 Laboratory of Aquaculture, Institute of Oceanography and Fisheries, Split, Croatia

**Keywords:** *Anisakis pegreffii*, In vitro culture, Larval development, SEM, TEM

## Abstract

**Background:**

Anisakiasis is a foodborne disease caused by the third-stage larvae (L3) of two species belonging to the genus *Anisakis*: *Anisakis pegreffii* and *Anisakis simplex* sensu stricto. Both species have been the subject of different -omics studies undertaken in the past decade, but a reliable in vitro culture protocol that would enable a more versatile approach to functional studies has never been devised. In nature, *A. pegreffii* shows a polyxenous life-cycle. It reproduces in toothed whales (final host) and disseminates embryonated eggs via cetacean faeces in the water column. In the environment, a first- (L1) and second-stage larva (L2) develops inside the egg, and subsequently hatched L2 is ingested by a planktonic crustacean or small fish (intermediate host). In the crustacean pseudocoelom, the larva moults to the third stage (L3) and grows until the host is eaten by a fish or cephalopod (paratenic host). Infective L3 migrates into the visceral cavity of its paratenic host and remains in the state of paratenesis until a final host preys on the former. Once in the final host’s gastric chambers, L3 attaches to mucosa, moults in the fourth stage (L4) and closes its life-cycle by becoming reproductively mature.

**Methods:**

Testing two commercially available media (RPMI 1640, Schneider’s *Drosophila*) in combination with each of the six different heat-inactivated sera, namely foetal bovine, rabbit, chicken, donkey, porcine and human serum, we have obtained the first reliable, fast and simple in vitro cultivation protocol for *A. pegreffii*.

**Results:**

Schneider’s *Drosophila* insect media supplemented with 10% chicken serum allowed high reproducibility and survival of adult *A. pegreffii*. The maturity was reached already at the beginning of the third week in culture. From collected eggs, hatched L2 were maintained in culture for 2 weeks. The protocol also enabled the description of undocumented morphological and ultrastructural features of the parasite developmental stages.

**Conclusions:**

Closing of the *A. pegreffii* life-cycle from L3 to reproducing adults is an important step from many research perspectives (e.g., vaccine and drug–target research, transgenesis, pathogenesis), but further effort is necessary to optimise the efficient moulting of L2 to infective L3.

**Graphical Abstract:**

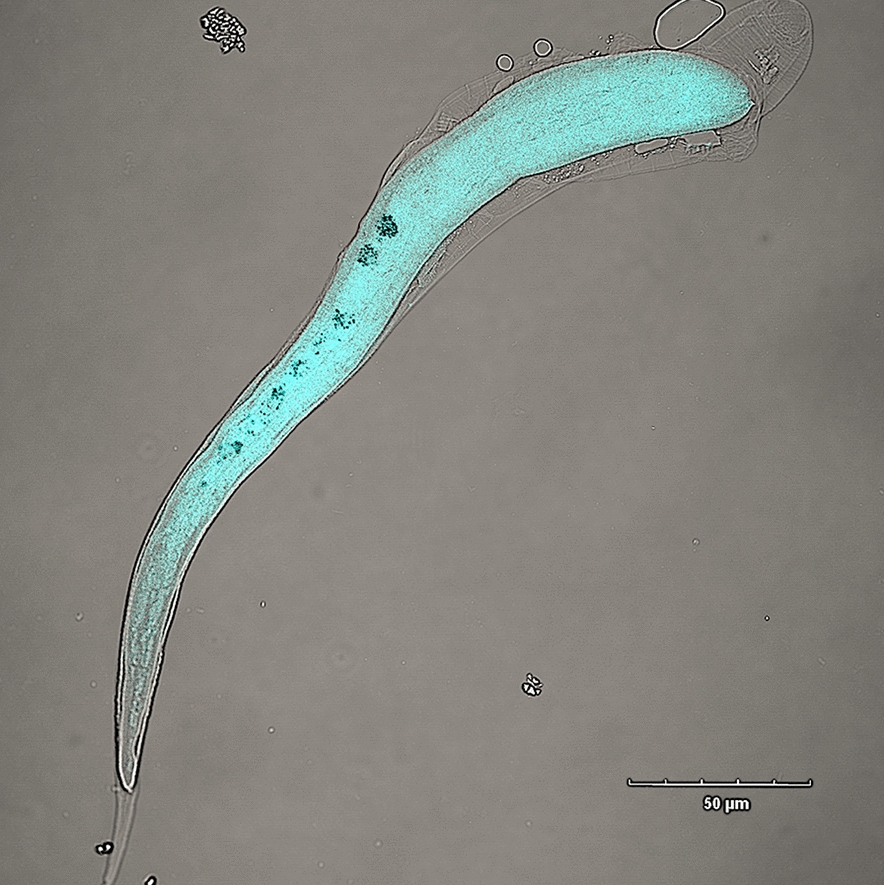

## Background

A baseline requirement for efficient downstream studies of helminths parasitic in livestock and humans is a reliable protocol for their in vitro culture, allowing fast and simple implementation, and a reproducible high survival/fecundity rate of at least the parental population. This is particularly important for -omics applications, where nematode fitness and physiological functionality under in vitro conditions should be carefully considered, as any oscillation in that respect is bound to reflect in the downstream data, leading to misinterpretation. While mortality in the control group maintained in vitro is an evident reason for repeating the trial, perturbation in reproductive success is less likely to be noticed, yet it implicates a deviation from the physiological status that could affect the measurements.

As causative pathogens of the emerging helminthiasis known as anisakiasis, two sibling species, *Anisakis simplex* sensu stricto (s.s.) and *Anisakis pegreffii*, have been a focus of different -omics research that has revealed new insights into host–parasite interactions, drug target discovery, functional ultrastructure and allergen identification [1–9]. These studies relied mainly on nematodes that were freshly isolated from the infected fish, which is limiting with respect to the availability of infected fish all year round, the time range when the experiment must be performed and the developmental stage to be used.

Although an *A. pegreffii *in vitro culture protocol has never been devised, researchers have relied on a protocol formulated for its sibling species *A. simplex* s.s., assuming equal efficiency for the two closely related species. Attempts at in vitro culture of *A. simplex* date back to the mid-twentieth century using formulations of “in-house”-prepared culture media and tissue extracts supplemented with different sera, yielding only fourth-stage larvae (L4) that failed to reach maturity (the timeline of different media used in the past is described by Grabda [10]). Banning [11] was the first to obtain mature nematodes using fresh bovine liver extract enriched in cattle blood and sodium citrate under pH 2 and 37 °C. Grabda [10] used the same liver extract and cattle blood-based protocol, but further lowered the pH to 1–1.5 with hydrochloric acid, achieving 50% survival and eggs discharged 23–29 days post-incubation. That study is the only one to document *Anisakis* developmental changes through schematic drawings and histological micrographs. Later work with the same protocol confirmed egg fertilisation after 29 days, but the survival rate decreased to 33.3% [12]. Iglesias et al. [13] optimised the in vitro cultivation, replacing liver extract and blood with a commercial cell culture media (RPMI 1640) supplemented with heat-inactivated foetal bovine serum (FBS; 20%), mimicking the final host gastric environment by the addition of 1% pepsin, pH 4, 37 °C and 5% CO_2_ in an air atmosphere. The authors obtained the adult stage from L4 after 16–57 days, with average survival of 91.3%. Despite a wide temporal range to reach the adult stage, this protocol has been established as the main tool for the cultivation of *A. simplex* s.s. intended for different downstream applications.

Prompted by repetitive failure to obtain a mature stage of *A. pegreffii* following the later in vitro cultivation protocol, we exposed freshly isolated *A. pegreffii* third-stage larvae (L3) to two commercially available media in combination with each of the six different heat-inactivated sera: foetal bovine, rabbit, chicken, donkey, porcine and human serum. The aim was to develop a reliable, fast and simple in vitro cultivation protocol that would allow high reproducibility and survival of *A. pegreffii*, and to describe undocumented morphological and ultrastructural features of parasite developmental stages.

## Methods

### *Anisakis* sp. isolation

*Anisakis* type I larvae were collected from the blue whiting *Micromesistius poutassou* caught by a commercial fisherman in the Adriatic Sea, stored overnight on ice and delivered the next morning to the Institute of Oceanography and Fisheries, Croatia. Vigorous and robust larvae actively migrating out of fish viscera were carefully removed with forceps, washed in a physiological saline solution and shipped in batches of 100 third-stage larvae (L3) in 50-ml tubes in filtered and autoclaved seawater by express courier to the Institute of Parasitology, Czechia. Less than 48 h elapsed from the host fishing until the placement of L3 in culture. Upon arrival, L3 were subjected to three consecutive washes in autoclaved M9 buffer with 1% penicillin/streptomycin (PS) (Sigma, P4333-20ML) and nystatin (Sigma, N9150-20ML), each of 30 min duration, at room temperature protected from light. L3 were then checked for morphological traits of type I larvae and cuticle integrity under a stereomicroscope (Zeiss, Stemi 305).

### Cell culture media preparation

For the culture of L3 isolated from fish, two commercially available media were used: RPMI 1640 with L-glutamine and sodium bicarbonate (Sigma, R8758-1L) for vertebrate cell culture and Schneider’s *Drosophila* (Gibco, 21720024) media for insect cell culture. Each medium was supplemented with 1% sodium pyruvate (Sigma, S8636-100 ML), 2% PS, 1 ml/l nystatin and liver concentrate (5 µg/ml) (Sigma, 2023-50G) and 10% of one of the six sera: heat-inactivated foetal bovine (FBS) (Sigma, F9665-500ML); rabbit (R) (Merck, R9133-10ML); chicken (C) (Merck, C5405-100ML); normal donkey (D) (Merck, S30-100ML); porcine (P) (Merck, P9783-500ML); and human type AB (male) (H) (Merck, H4522-20ML). Finally, 0.001% pepsin (Sigma, 1071850100) was added to the media, pH was adjusted to 4 by 1 M HCl, and the complete media was syringe-filtered (0.22 µm) and stored at 4 °C until further use. A 5 ml aliquot was left in a sterile tube at 37 °C to show for any potential media contamination.

When adult parasites were obtained following the above protocol, eggs were collected and incubated (see below) to harvest hatched larvae. For the culture of the latter, three commercially available media were used: Dulbecco’s modified Eagle’s medium–low-glucose (Dulbecco’s modified Eagle medium [DMEM] lg) (D5523-10X1L), DMEM–high-glucose (DMEM hg) (D5648-10X1L) and Schneider’s *Drosophila.* Each medium was supplemented with different concentrations of the chicken serum (10, 20 or 50%), 2% PS and 1 ml/l nystatin, and with or without 5 M NaCl to increase media osmolality to approximately 400 mOsm/kg. Development in autoclaved seawater was used as a negative control.

### In vitro culture, egg harvesting and culture of second-stage larvae (L2)

A single L3 was placed per well with 1 ml of each filtered complete media in a 24-well plate and incubated at 37 °C (to mimic final host’s body temperature) with a 5% CO_2_ atmosphere. Media was changed twice a week throughout the experiment. Larvae were checked under a stereomicroscope (Zeiss, Stemi 305), and dead larvae were collected and counted daily. On the 14th day of incubation, larvae from a single well plate cultivated under the same conditions were pooled in the autoclaved, 50-ml conical-bottom centrifuge tube (Merck, CLS430828) with 30 ml of complete media. Tubes with loosely closed lids were placed back in the incubator. This was done to allow for more natural mating conditions, a higher quantity of nutrients, and prevention of escape from the 24-well plate. Media was checked for eggs at changing times (2×/week) under the microscope after the third week.

In week 3, the released eggs were observed in the media and adults were gently placed with blunt tweezers in a new 50-ml tube with fresh media. The next day, the fresh media was used to collect eggs released over the previous 24 h by centrifugation at room temperature, 16.1 g for 20 min. The supernatant was discarded, and the pellet was washed twice with 10 ml of autoclaved seawater by centrifugation at 16.1 g for 20 min. Eggs were resuspended in the seawater by gentle shaking and sieved through DNase/RNase-free cell strainers with 70 µm nylon pore size (Corning, 734-2761) to remove organic detritus, and 1 ml of the suspension was redistributed in 24-well plates and held at 17 °C (to mimic seawater temperature) and 5% CO_2_ atmosphere.

Hatched larvae were collected by aspiration using a 100 μm cut tip on the micropipette and redistributed in six-well plates with different media. Larvae were checked daily under the microscope (Olympus, CKX53), and the media was changed by aspiration twice a week. Cultures with adults were also monitored daily under the stereomicroscope (Zeiss, Stemi 305), and mortality was noted and plotted in R using ggplot2 [14] and ggthemes. The experiment was carried out five times.

### Molecular identification of *Anisakis* sp. from in vitro culture

A subsample of 13 adults, two L4 and four egg suspensions was used for molecular identification of cultured *Anisakis* sp. DNA was extracted by SSTNE [spermidine/spermine/tris/sodium chloride/EDTA (ethylenediaminetetraacetic acid)/EGTA (egtazic acid)] buffer and the salt precipitation method according to a previously reported method [15]. A portion of cytochrome oxidase subunit 2 (*cox2*) was amplified from 50 ng of purified DNA, by combining 2 U of FastGene^®^ Taq Polymerase (Nippon Genetics Europe GmbH), 2.5 mM MgCl_2_, 0.2 mM dNTP and 0.4 mM of each primer, forward 211F 5′-TTTTCTAGTTATATA GATTGRTTYAT-3′ and reverse 210R 5′-CACCAACTCTTAAAATTA C-3′ [16], with annealing temperature set at 46 °C. PCR products were checked in 1% agarose gel and sequenced commercially (Macrogen Europe Laboratory, Netherlands). Obtained sequences were aligned with available *Anisakis* spp. sequences available in GenBank (https://www.ncbi.nlm.nih.gov/genbank/) by ClustalW implemented in MEGA X software [17], checked in BioEdit and manually corrected for erroneously read bases. For species identity, sequences were compared to those available in GenBank using BLASTn [18]. Sequences were deposited in GenBank with accession numbers OP620697–OP620715.

The same samples were genotyped using the PCR-based restriction fragment length polymorphism (RFLP). Samples were amplified at the internal transcribed spacer (ITS) locus as described above with the forward primer BD1 5′-GTCGTAACAAGGTTTCCGTA-3′ [19] and reverse primer BD2 5′-TATGCTTAAATTCAGCGGGT-3′ [20], and annealing temperature at 54 °C. Subsequently, PCR products were digested with 5 U of HinfI restriction endonuclease (Promega, USA) and visualised in 2% agarose gel, and species identified according to the RFLP pattern as reported by D'Amelio et al. [21].

### Morphological characterisation of *A. pegreffii* developmental stages

#### Cryo- and conventional scanning electron microscopy (SEM)

Specimens (*n* = 3 per developmental stage) of a late L4 and early adult from week 5 of cultivation were thoroughly washed in phosphate-buffered saline (PBS) and fixed in cold 2.5% glutaraldehyde in PBS overnight. The anterior and posterior parts of the specimens were cut, postfixed in 2% OsO_4_ and dehydrated in ascending concentrations of acetone, with 5 min incubation at each step. Samples were then critical point-dried (CPD2, Pelco TM), mounted on aluminium stubs using carbon conductive tape and sliver paste, and coated with gold (SEM coating unit E 5100, Polaron). Anterior and posterior parts of L4 and adult *A. pegreffii* were observed in a field emission scanning electron microscope (JEOL JSM-7401F) operating at 0.1–30 kV.

To document vesicles discharged from the excretory pore that was observed in the above collected specimens by conventional SEM, another pair of L4 and adult from the same batch were frozen by plunging in liquid nitrogen slush. Specimens were first oriented onto an aluminium SEM specimen holder covered with Tissue-Tek^®^ (EMS), then transferred under vacuum to a Cryo ALTO 2500 chamber of a JEOL 7401F SEM, and cooled at −140 °C. Inside the chamber, sublimation was conducted at −95 °C for 5 min. The temperature was then decreased to −140 °C, and the samples were sputter-coated with platinum-palladium for 100 s. Finally, samples were examined using the JEOL 7401F SEM at 1–2 kV using the Everhart–Thornley detector of secondary electrons.

#### Confocal microscopy

Newly hatched larvae (0 h) and larvae at 12, 24, 48, 72 h, and 1 and 2 weeks post-hatching were fixed in 5% glacial acetic acid in ethyl alcohol for 10 min, washed twice in PBS and then permeabilised in PBS with 1% Triton X-100 for 10 min. After washing three times in PBS, larvae were mounted on standard microscope slides, covered by DAPI (4′,6-diamidino-2-phenylindole) shield and coverslip, and sealed with nail polish. Specimens were observed under a confocal microscope (Olympus FV3000).

#### Transmission electron microscopy (TEM)

Two female *A. pegreffii* were dissected under the stereomicroscope, and samples of the uterus and oviduct were collected in cold PBS and immediately processed using high-pressure freezing and freeze substitution as described earlier [3]. After dehydration in ascending concentrations of acetone (3×/15 min each concentration), samples were infiltrated for 1 h in 25, 50, and 75% mixtures of low-viscosity Spurr resin (SPI Chem, West Chester, PA, USA) and anhydrous acetone, left overnight in 100% resin, transferred to embedding moulds and polymerised for 48 h at 60 °C. Semi-thin sections (0.5 μm) stained with 1% toluidine blue were observed under a light microscope for orientation. Ultrathin sections (0.07 μm) mounted on Formvar-coated single-slot grids were contrasted in ethanolic uranyl acetate (30 min) and lead citrate (20 min) and observed under a JEOL JEM-1400 microscope (JEOL, Akishima, Tokyo, Japan) operating at an accelerating voltage of 120 kV. Images captured with a XAROSA 20-megapixel CMOS camera (EMSIS GmbH) were assembled and annotated in Inkscape 1.0 software (https://inkscape.org).

## Results

### Molecular identification of *Anisakis *sp. from in vitro culture

Based on the BLASTn results for the *cox2* sequences, all samples were identified as belonging to *A. pegreffii*, sharing 99.66–100% sequence identity with sequences deposited in GenBank. RFLP analysis of the ITS region resulted in two distinct patterns: the first with three fragments of approximately 370, 300 and 250 bp corresponding to *A. pegreffii*, and the second with four fragments of approximately 320, 370, 300 and 250 bp corresponding to recombinant genotype or putative hybrids. In total, 16 samples (84.21%) were assigned to *A. pegreffii*, and three samples of adults (15.79%) were identified as belonging to the recombinant genotype.

### Culture of* A. pegreffii*

**Fig. 1:**
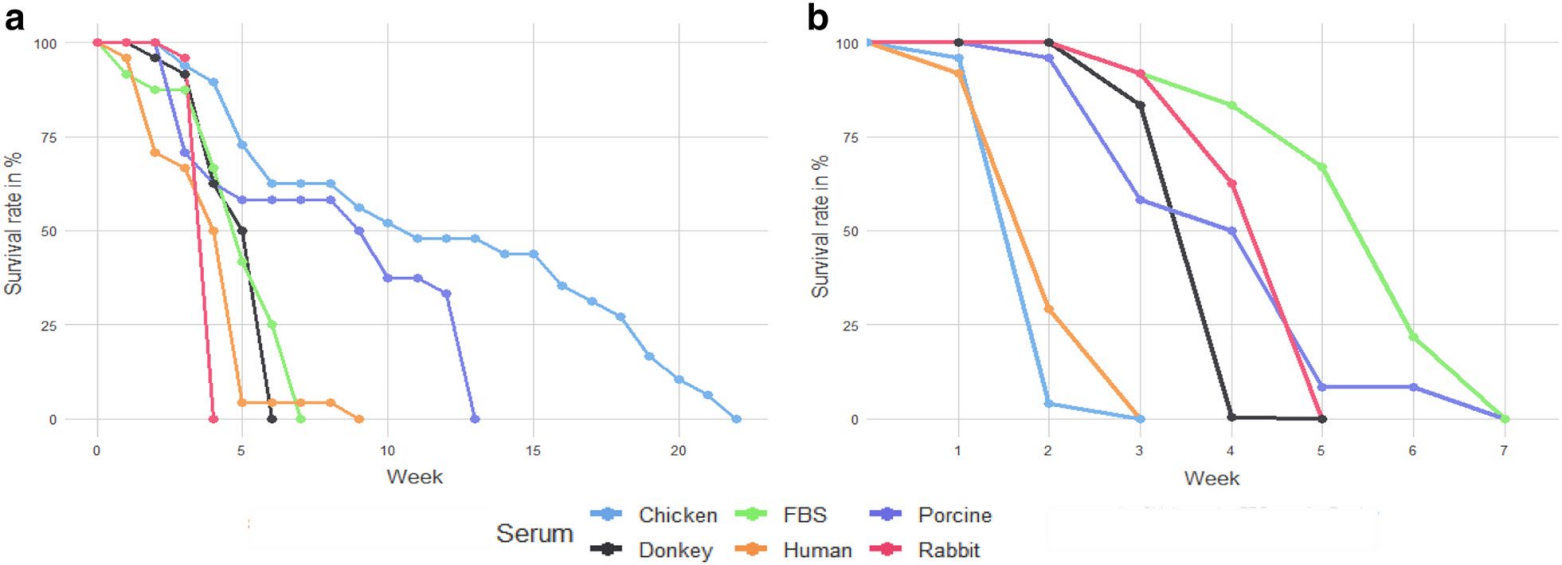
Survival rate (*y*-axis, %) of *A. pegreffii* cultured in vitro in **a** Schneider’s *Drosophila* media and **b** RPMI 1640 supplemented with 10% chicken, foetal bovine, porcine, donkey, human and rabbit serum, over time (*x*-axis, weeks)

Infective L3 of *A. pegreffii* reached the fertile adult stage only in Schneider’s *Drosophila* media supplemented with 10% chicken serum (Fig. 1). L3 moulted into L4 on the fourth day of incubation in the culture media, recognisable by the conspicuous zig-zagged intestine, abundant shedding of the cuticle and loss of the prominent mucron. L4 showed an increase in length and thickness and body colouring during the second week in a culture that suggested transit through the pre-adult (L5) stage towards maturation. The adult stage was reached at the beginning of the third week in culture, characterised by a wider anal region and the development of a spicule in males. Female winding loops of the oviduct were not distinguishable by the naked eye from the male seminal ducts without specimen dissection. The oviposition peaked in the sixth week of culture and continued to increase until the 16th week of culture. Even though the oviposition continued further, a lower egg yield and fertilisation rate were observed.

The culture of the nematode in Schneider’s *Drosophila* media supplemented with other sera showed consistent failure, except when 10% of porcine serum was used. However, the results were inconsistent, since adulthood was reached in the fourth and seventh week, and the experiment was terminated in the 13th and 9th week, respectively. Interestingly, while oviposition was observed, eggs were found to be unfertilised, and no development was observed.

The addition of rabbit, human and donkey serum and FBS in Schneider’s *Drosophila* media all aided in moulting of L3 to L4, but larvae died in the third, fourth, sixth and seventh week in culture, respectively. The culture of L3 in RPMI 1640 media supplemented with sera did not prove to be successful in terms of reaching the adult stage and oviposition. L3 moulted in L4 in 4 days as in insect media but never transited in the pre-adult stage. The survival rate declined over time, and the culture was terminated in the third, fifth and seventh week in RPMI 1640 supplemented with chicken and human sera, donkey and rabbit sera, and porcine serum and FBS, respectively.

### Hatching of *A. pegreffii*

**Fig. 2:**
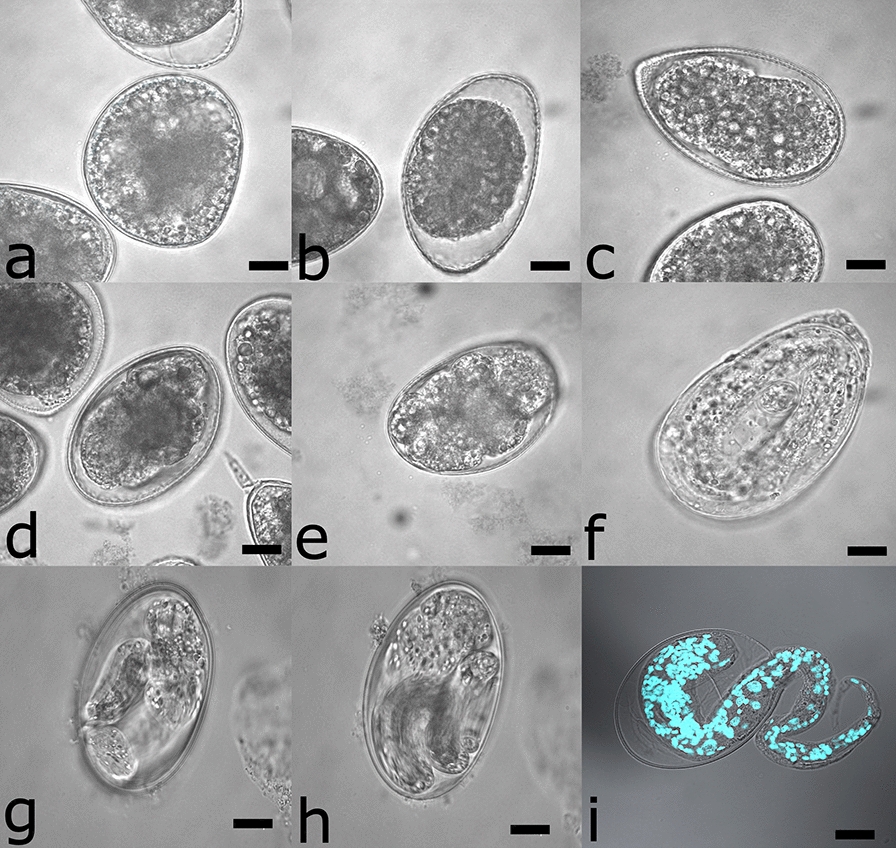
Eggs harvested from in vitro culture of *A. pegreffii* and incubated in seawater at 17 °C in 5% CO_2_ atmosphere for 6 days before hatching of L2. Incubation time up to **a** 12 h, **b** 24 h, **c** 48 h, **d** 72 h, **e** 96 h, **f** 120 h, **g** and **h** 144 h, and **i** 168 h, resulting in the L2 hatching from the egg. Note the wrinkled ensheathment of the L1 cuticle. Nuclei of hatching L2 are stained by DAPI. Scale bars: 10 µm

Freshly oviposited eggs (40–60 µm in perimeter) have negative buoyancy, are spherical to mildly ovoid, with a moderately thick eggshell, composed of three layers: the innermost thin layer, the mid-thickness and translucent layer, and the outermost thin layer (Fig. 2). The space is fully occupied by acellular, densely granulated material. In 24-h-old embryonated egg, cell cleavage takes place, leaving more perivitelline space for the gastrulation stage. The first kidney bean-shaped L1 appears between 48 and 72 h, growing in size and occupying most of the perivitelline space. Approximately 24–48 h before hatching, vermiform larvae with a single tight sheath of the cuticle are very motile, with erratic and convoluted movements widespread within the egg. Prior to hatching, the boring tooth is visible, as well as the second cuticular sheet of the L2. The eclosion of L2 happens mostly on the sixth day at 17 °C, by active thrusting of the larval head on the targeted eggshell wall that lasts for a couple of hours. Pharyngeal pumping is observed 24 h before hatching. Newly hatched L2 enveloped in L1’s cuticle has a “tadpole” appearance, with wide cephalic parts that decrease aborally towards the tail tip, the latter bearing small mucron. L2 have negative buoyancy and show erratic movement consisting of simultaneous “flicking” of the opposite body ends.

Marking of the cell nuclei by DAPI showed the signal more abundant in the anterior oesophageal area, continuing laterally in the second third of the body, enveloping the pseudocoelom (body wall), and centrally (intestine). Dark and finely granulated material is observed occasionally inside the last third of the intestine. L2 grow in length over the first 72 h, and at this stage in their mid-portion, a cell with a more dispersed DAPI signal in a larger nucleus is observed. The signal is more conspicuous in 1-week-old L2.

**Fig. 3:**
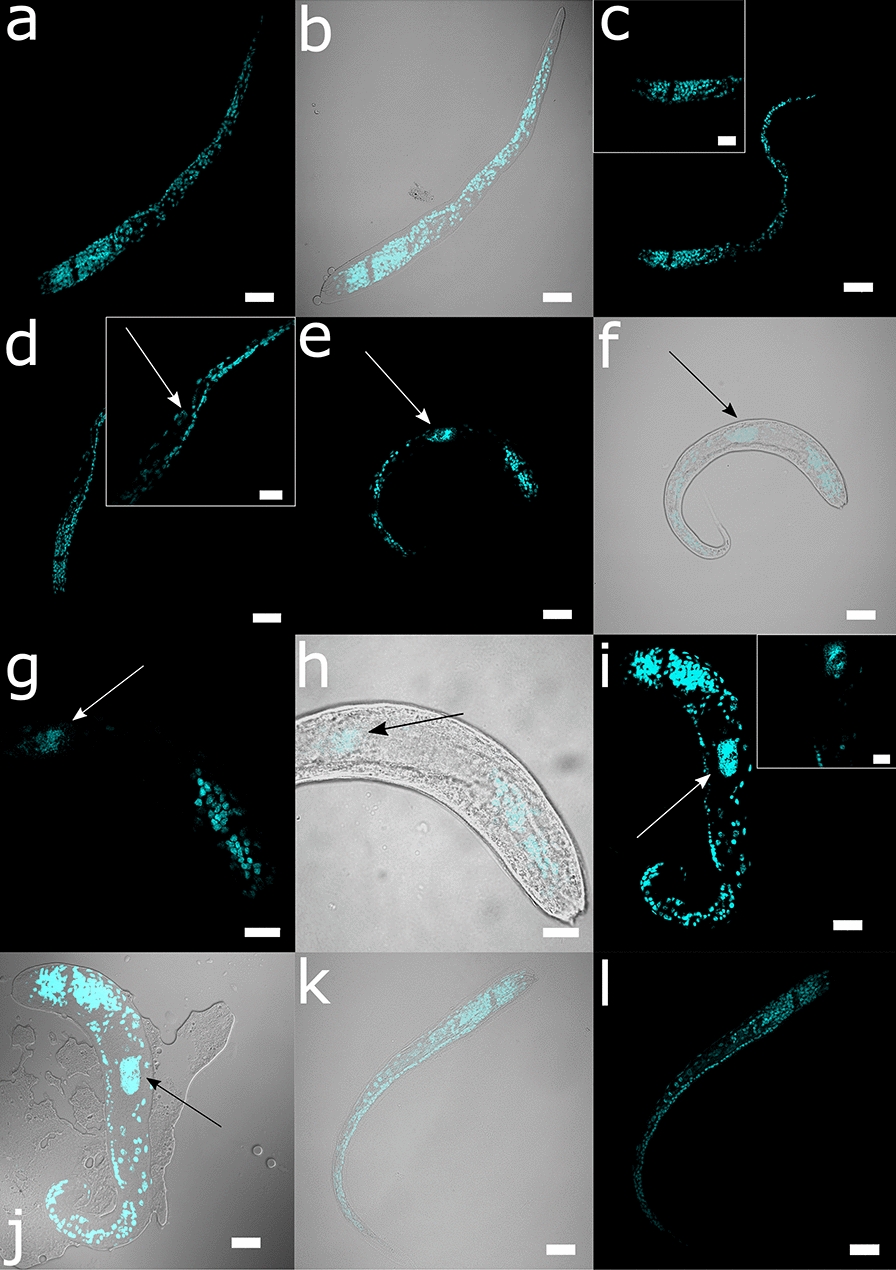
*Anisakis pegreffii* L2 hatched in vitro and cultured up to 24 h (**a, b**), 48 h (**c**), 72 h (**d**), 1 week (**e–h**) and 2 weeks (**i, j**) in Schneider’s *Drosophila* media, and 2 weeks in seawater (control; **k, l**). DAPI (blue) is localising larval nuclei. Insets show details under higher magnification: (**c**) no large-nucleus cell attributable to the excretory gland cell is observable; **d** the first appearance of a larger-nucleus cell; and **e-i** presumable excretory gland cell. Note the increase in DAPI intensity in this cell through larval growth. White and black arrows indicate presumable excretory gland cell in fluorescence and bright-field pictures, respectively. Fluorescence is overlaid on the complementary bright field for better orientation (**a** and **b**; **e** and **f**; **g** and **h**; **i** and **j**; **k** and **l**). Scale bars: 10 µm (**g**, **h**, and insets in **c**, **d** and **i**); 20 µm (the rest)

L2 grew well in all tested media (DMEM low-glucose, DMEM high-glucose, Schneider’s *Drosophila*) supplemented with a higher concentration of chicken serum (20 and 50%), becoming thicker compared to control L2 grown in seawater (Fig. 3). Mortality was not counted and the difference between media was not statistically tested, because the culture in six-well plates and change of culture media by aspiration was found to be time-consuming, and many L2 were used for microscopy. Although L2 grown in culture media showed fast development, the dead larvae were usually observed enveloped in L2 cuticle.

### Morphological traits of *A. pegreffii*

**Fig. 4:**
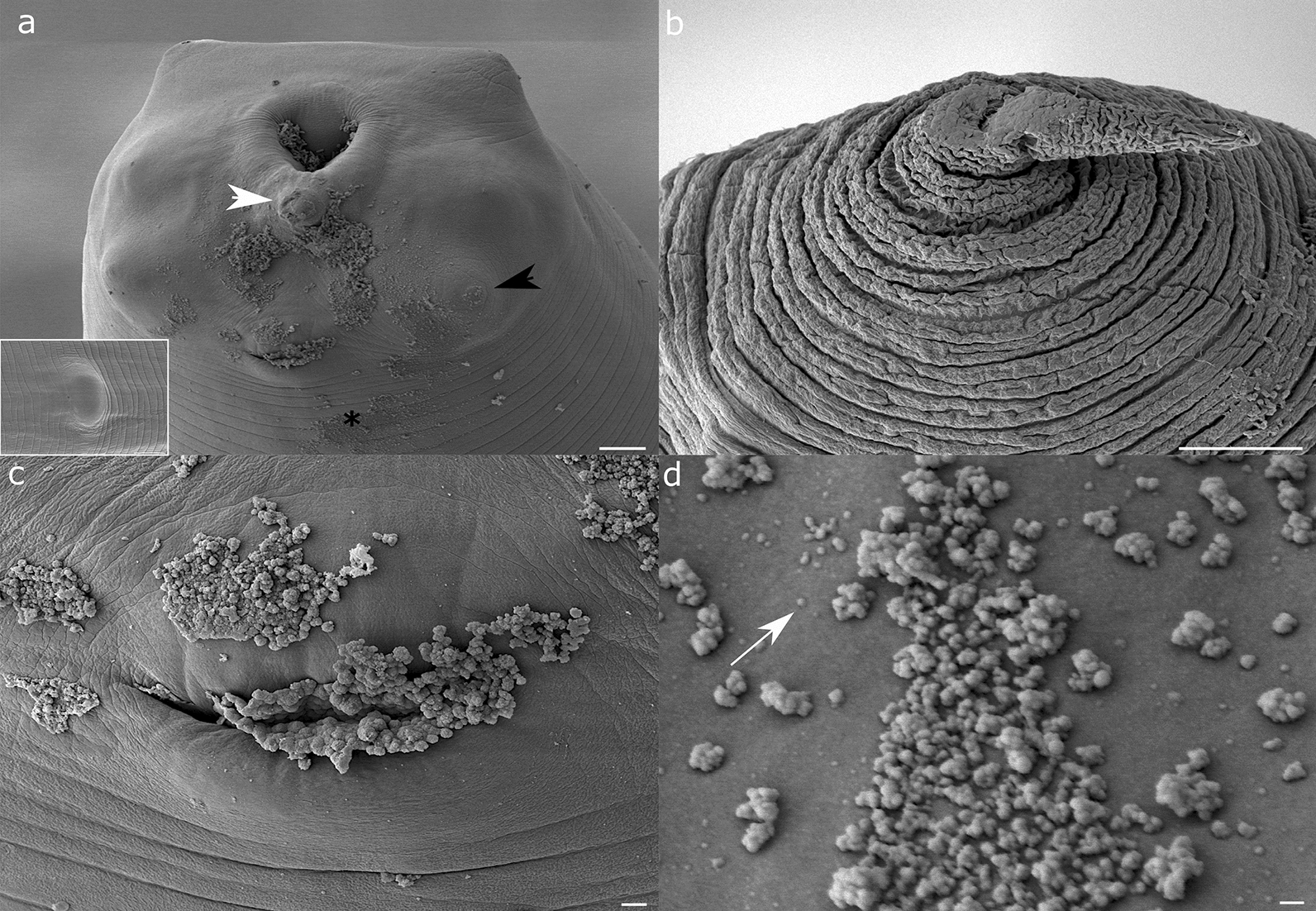
Representative scanning electron microscopy (SEM) and cryoSEM renderings of *A. pegreffii* L3: **a** Anterior region with prominent spherical oral opening surrounded by the three inconspicuous lips. White arrowhead shows the boring tooth and black arrowhead the cephalic papilla on the ventrolateral lip. Inset: phasmid on the upper body surface. **b** Mucron on the posterior region, surrounded by wrinkles of L3 cuticle. Note that the mucron has been damaged in the process. **c** Excretory pore masked by discharge from the excretory gland cell. **d** Higher magnification of the excretory pore surface showing extracellular vesicles in clusters or individually (white arrow). Scale bars: 10 µm (**a, b**); 1 µm (**c**); 100 nm (**d**)

**Fig. 5:**
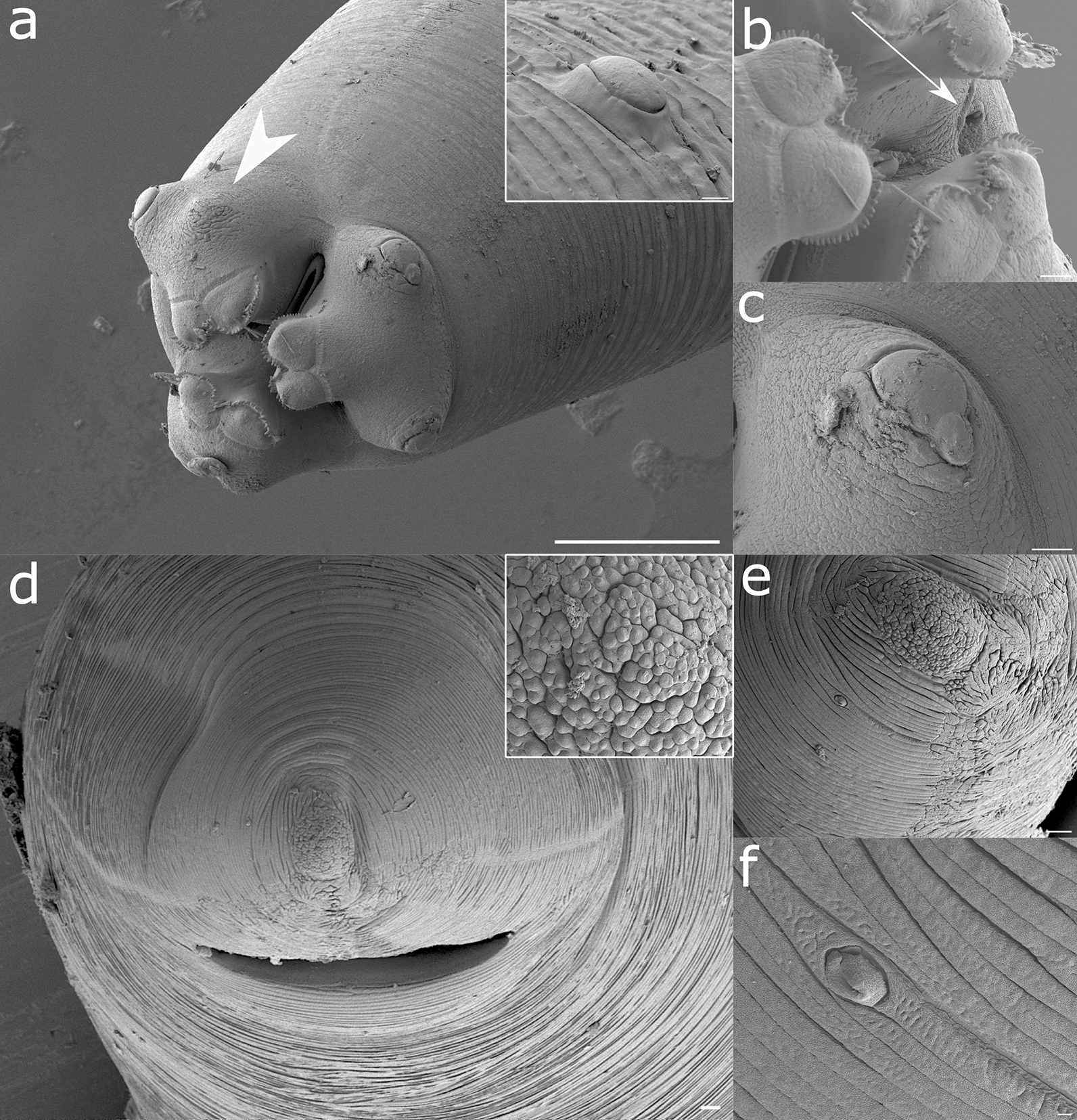
Representative scanning electron microscopy (SEM) and cryoSEM renderings of *A. pegreffii* L4: **a** Anterior region with three prominent lips shielding a tricuspid oral opening. White arrowhead shows the amphid on the lip, and the deirid in the inset. **b** Higher magnification of the lips. White arrow shows excretory pore. **c** Cephalic papilla on the dorsal lip. **d** Bird’s-eye view of the posterior region with transversely located slit-like anal opening. Inset: mulberry-like cluster of cells located on the tail tip. **e** Lateral view of the posterior part showing one of the two phasmids, and no distinct caudal papillae. **f** Phasmid set laterally from the tail tip. Scale bars: 100 µm (**a**); 10 µm (inset **a**, **b, c, d, e**); 1 µm (inset **d** and **f**)

**Fig. 6:**
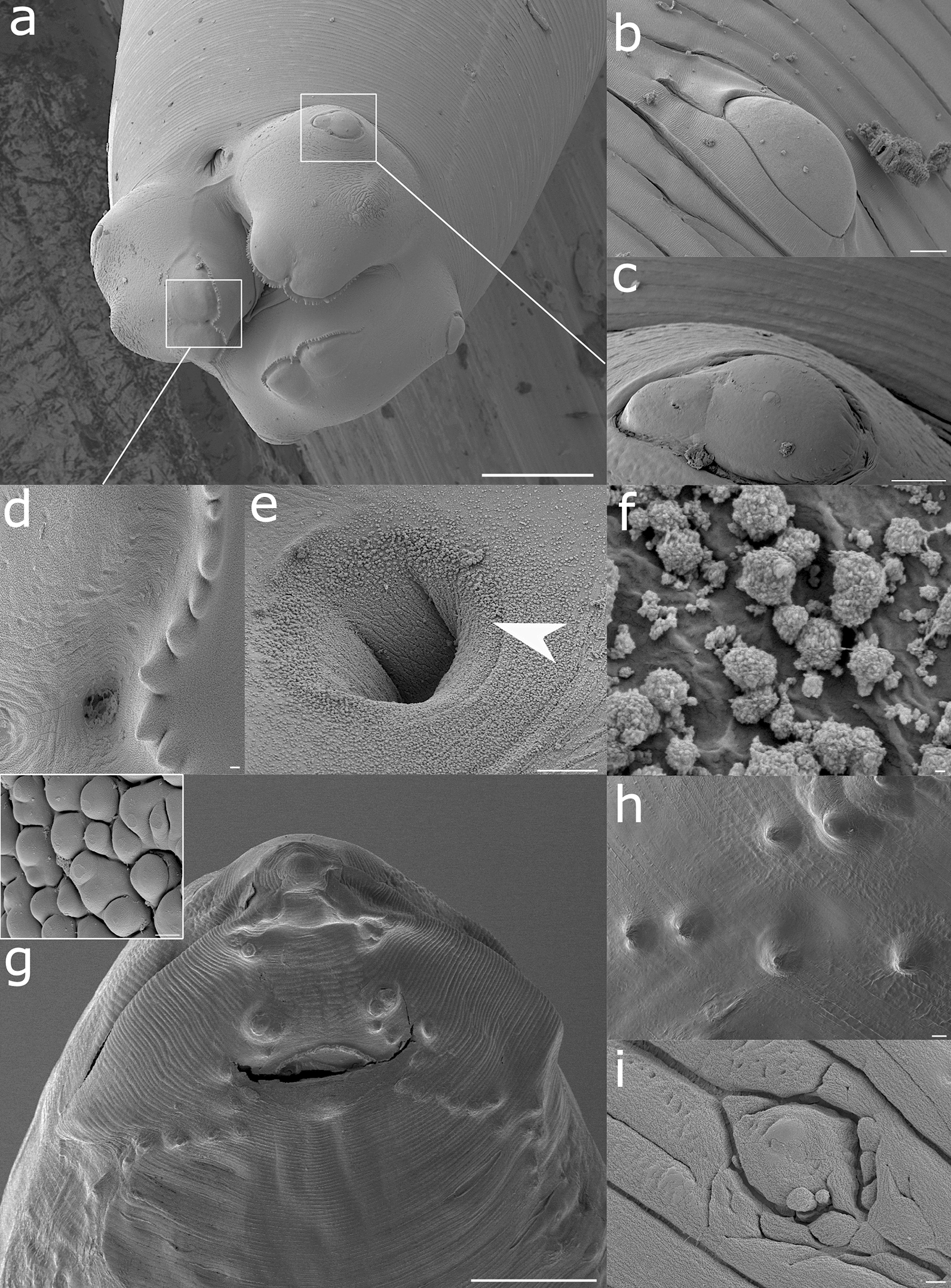
Representative scanning electron microscopy (SEM) and cryoSEM renderings of *A. pegreffii* female: **a** Anterior region with three prominent lips shielding a tricuspid oral opening. White frames depict the area of **c** cephalic papilla and **d** serrated lip edge and a foramen in the center (**d**) at higher magnification. **b** Dereid. **e** Excretory pore with scattered discharge on its rim (white arrowhead). **f** Clusters of aggregated extracellular vesicles on the surface of the excretory pore. **g** Frontal view of the posterior region with transversely located slit-like anal opening and rows of caudal papillae descending from the tail tip downward. Inset: mulberry-like cluster of cells located in the tail tip. **h** Detail of caudal papillae. **i** Phasmid set laterally from the tail tip. Scale bars: 100 µm (**a, g**); 10 µm (**b, c, e, h**); 1 µm (**d**, inset **g** and **i**); 100 nm (**f**)

Ontogenetic changes are noticeable in the anterior oral and posterior caudal regions of the nematode (Figs. 4, 5, 6). In the former, three lips (dorsal, right and left lateroventral) increase in size and robustness, closing over the tricuspid mouth. Excretory pore observed by cryoSEM reveals that all developmental stages (i.e., L3, L4 and adults) show discharge appearing as raspberry clusters composed of smaller and larger vesicles (size range ~ 50–210 nm). Sensory elements on the nematode head (cephalic sensory papillae and amphids) and body (deirid) become prominent, while the boring tooth is lost after moulting in L4, the same as the mucron on the posterior part. A mulberry-like cluster of cells present on the tail tip shows shedding from their surface. Two phasmids are placed lateral to the tail tip. Anal papillae are observed in late L4 specimens, descending from the tail tip to a slit-like anal opening and further slanting away from it to the lateral plane.

**Fig. 7:**
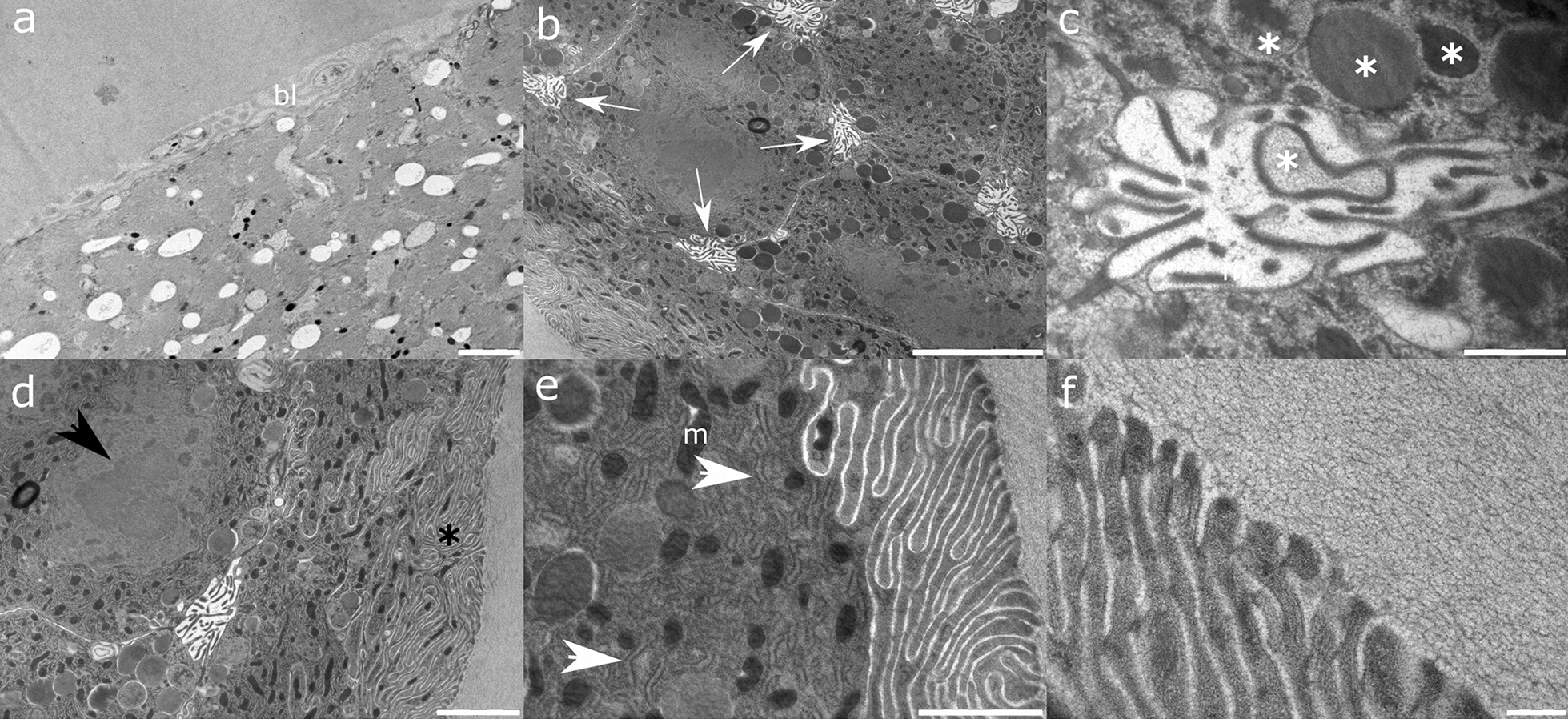
Representative transmission electron micrographs of *A. pegreffii* uterus formed by **a** basal lamina (bl) lined with a thick multicellular layer of sheath cells abundant in myofibril bundles. Note rare, scattered electron-dark mitochondria and more abundant electron-light granules. **b** Cuboid epithelial cells (in transverse section) with an electron-dense cytoplasm and microvilli localised on four cell edges interconnected in an intercellular space (white arrows). **c** Epithelial cells excreting electron-dense granules (putative vitellus) within the intercellular space. Note the different level of depletion of the granule content (white asterisk). **d** A layer of epithelial cells with labyrinth-like microvilli projecting in the uterus lumen (black asterisk). Note the three-leaf clover appearance of the prominent nucleolus (black arrowhead). **e** Apical periphery of the epithelial cell cytoplasm with electron-dark mitochondria (m) and intricate rough endoplasmic reticulum (white arrowheads). **f** Epithelial cell microvilli with a bilayer plasmalemma and electron-dense periphery. Scale bars: 5 µm (**b**); 2 µm (**a**, **d**); 1 µm (**e**); 500 nm (**c**); 200 nm (**f**)

Ultrathin sections of *A. pegreffii* uterus (Fig. 7) suggest a thick muscular multicellular layer providing support to densely packed large cuboid epithelial cells. Neighbouring epithelial cells at their four lateral sides form intercellular spaces with the intensive secretion of large electron-dense granules mediated through microvillous projection. Dense labyrinth-like microvilli are present on the cell apical surface. The cytoplasm has an electron-dense appearance due to abundant rough endoplasmic reticulum, dark and light cristate mitochondria and electron-dense granules. The nucleus is large and of irregular shape, with a prominent large nucleolus of three-leaf clover shape and scattered heterochromatin.

**Fig. 8:**
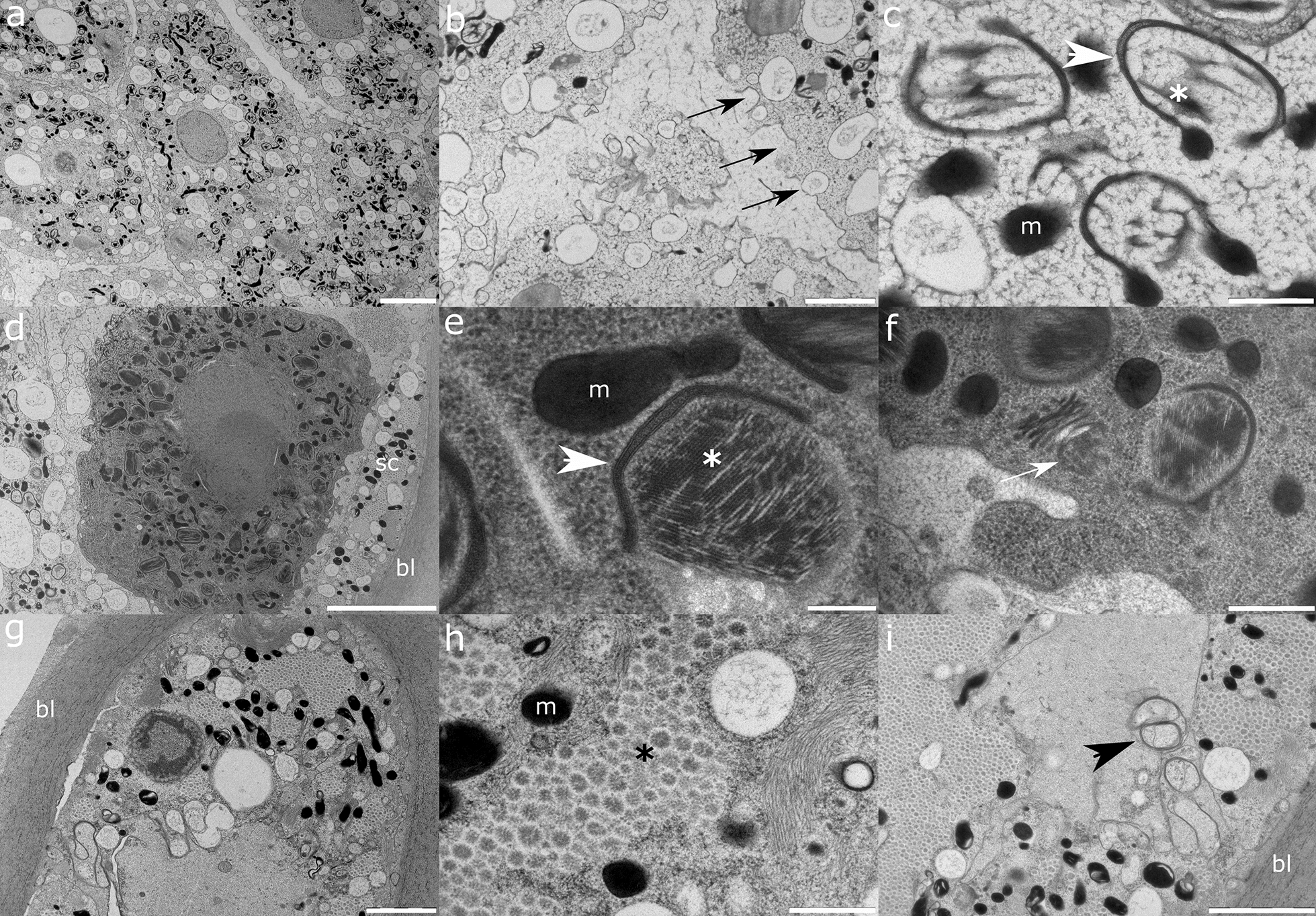
Representative transmission electron micrographs of *A. pegreffii* proximal oviduct encompassing the following: **a** Late oocytes with many large electron-light and depleted granules, small spherical and depleted refractile bodies, and electron-dense, vermiform mitochondria. Note a central large nucleus with prominent nucleolemma. **b** Oocytes excreting a lipid/proteinaceous substance in the oviduct lumen from its electron-light granules (black arrows). **c** Refractile bodies enveloped by a double membrane of the Golgi apparatus (white arrowhead) with depleted fibrillar content (white asterisk), and mitochondria (m). **d** Electron-dense oocytes with central nucleus and nucleolus, lacking a prominent nucleolemma. Note denser refractile bodies in its cytoplasm and lack of electron-light large granules. These rarely occurring cells were found in close contact via short pseudopodia to the sheathe cells (sc) in the basal lamina (bl). **e** Refractile body filled with fibrillar electron-dense material (in transverse section; white asterisk) enveloped in a membrane of the Golgi apparatus (white arrowhead), in proximity of a mitochondrion (m). **f** Periphery of the electron-dense oocyte with a pseudopodial projection and Golgi apparatus forming a refractile body (white arrow). **g** Cross section of the oviduct basal lamina (bl) with a single-cell layer of sheath cell. Note the spherical nucleus and large electron-light granules. **h** Bundles of myofibrils in cross section (black asterisk) and longitudinal section, surrounding a mitochondrion within a sheath cell. **i** Conspicuous multi-lamellar bodies (black arrowhead) forming in the cytoplasm of a sheath cell lining the basal lamina (bl). Scale bars: 5 µm (**a, b, d**); 2 µm (**g, i**); 500 nm (**c, h, f**); 200 nm (**e**)

The proximal oviduct (Fig. 8) shows basal lamina layered by a single layer of sheath cells, abundant with myofibril bundles. Late oocytes show electron-light cytoplasm abundant in the Golgi apparatus and large granules with depleted lipid/proteinaceous content expelled in the intercellular space, as well as fibrillar refractile material enveloped but not fully enclosed in membranes of the Golgi apparatus. Electron-light granules can be observed forming a bubble-like structure between the inner and outer nuclear membrane. Ribosomes and mitochondria are scarce, the latter elongated and electron-dense with no discernible cristae. The nucleus is large and spherical, with a prominent nucleolus. Parietally, rare and conspicuously electron-dense oocytes are observed. They are distinctive for abundant free ribosomes and Golgi apparatuses that produce electron-dense fibrillar material packed in double-layered membranes. They present a more electron-dense nucleus with non-discernible nucleolemma, while plasmalemma forms short pseudopodia.

**Fig. 9:**
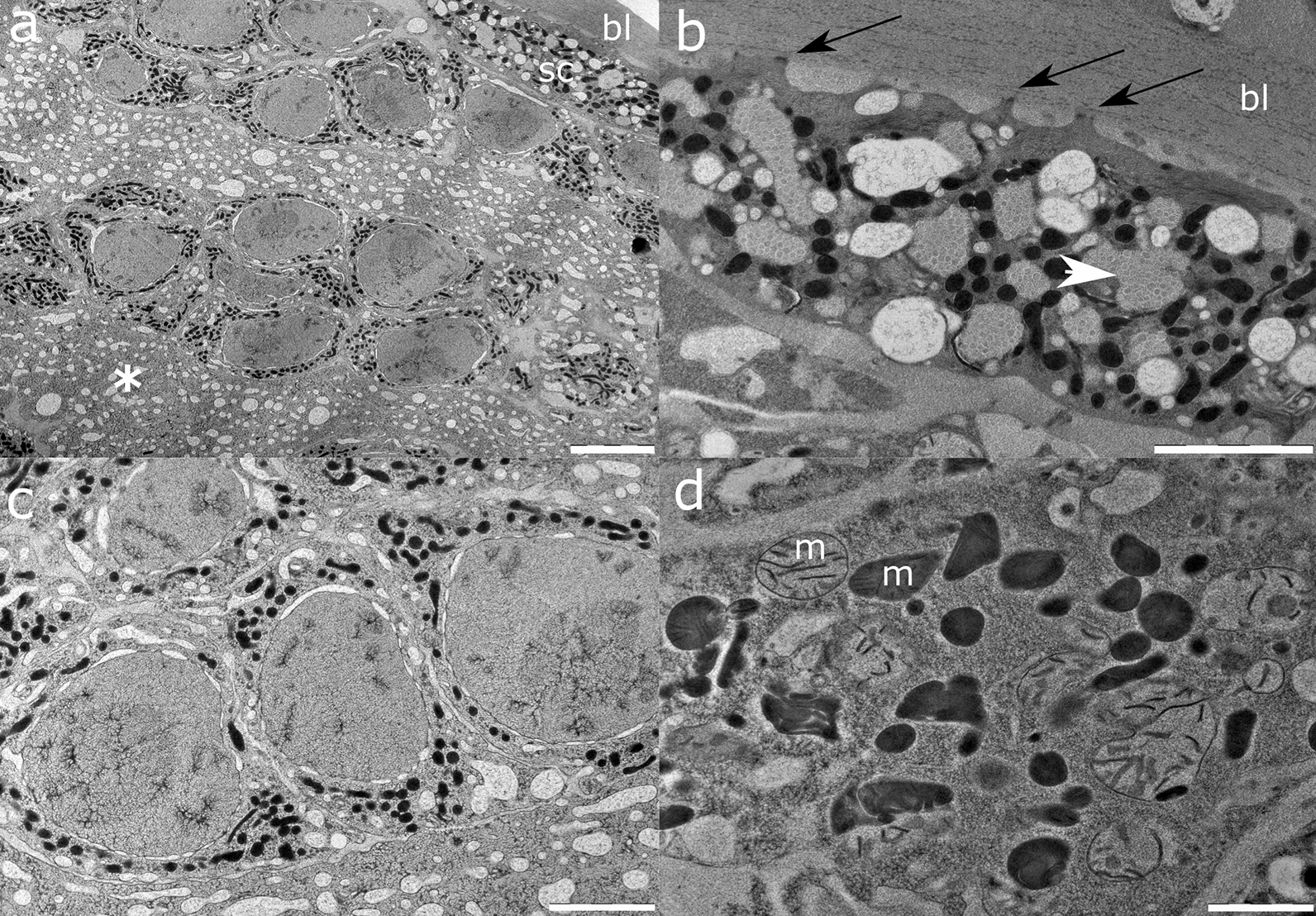
Representative transmission electron micrographs of *A. pegreffii* distal oviduct encompassing **a** clusters of early oocytes within the oviduct lumen filled with lipid/proteinaceous matter (white asterisk). Note sheath cells (sc) lining the basal lamina (bl); **b** myofibril bundles in the sheath cell (white arrowhead), attached with plasmalemma’s projections (black arrows) to the basal lamina (bl); **c** early oocytes with a prominent nucleus and nucleolus, and scarce cytoplasm abundant in electron-dense mitochondria; **d** early oocyte cytoplasm showing electron-dense and electron-light mitochondria (m)

The distal oviduct (Fig. 9) is lined by sheath cells similar in appearance to those found in the proximal oviduct. Sheath cells lack abundant large multi-lamellar bodies and electron-light granules with proteinaceous content, in contrast to those observed in the proximal oviduct. However, the oviduct lumen is filled with lipid/proteinaceous secretions originating from oocytes. These exhibit a prominent nucleus and nucleolus, and scarce cytoplasm abundant in electron-dense and electron-light mitochondria. Refractile bodies and electron-light granules are not observed at this stage in the oocyte cytoplasm.

## Discussion

Schneider’s *Drosophila* medium supplemented with 10% chicken serum enabled consistent closure of the *A. pegreffii* life-cycle from the L3 stage isolated from fish visceral cavity. Originally designated for the rapid growth of *Drosophila melanogaster* cell lines, this extremely nutritious insect media is also used for the culture of cell lines derived from other dipteran species. The reason that it better supports nematode development relative to vertebrate media might be related to the fundamental feature shared by insects and nematodes—ecdysis, or “skin shedding” during ecdysozoan development. While insects and nematodes descend in two very different phyla characterised by striking anatomical differences and evolution of specialised structures such as wings in the former, both are encompassed within the superphylum whose affiliates undergo moulting during development [22]. In fact, ecdysteroids or insect moulting hormones occur in nematodes in low concentrations, stimulating their moulting and affecting their reproduction [23]. Although insect medium was previously used to reach the nematode adult stage [24], it has been replaced by media for vertebrate cell lines, such as RPMI 1640 [13]. The latter enabled the maturation of adult *A. simplex* and *Hysterothylacium aduncum*, although through an inconsistent time span in the former, and provided no further development of hatched larvae of either species [25]. Time-wise, *A. pegreffii* developed in insect medium in a similar manner to *H. aduncum*, the difference being in the time necessary to reach L4 (4 days in *A. pegreffii* vs 2 weeks in *H. aduncum*), but the timing of the oviposition coincided (22–24 days in *A. pegreffii* vs 26 days in *H. aduncum*) [24].

However, we observed that insect media supplemented with FBS blocked *A. pegreffii* maturation, failing to support its late-stage development. This indicates inadequacy of the serum for this nematode, possibly related to its content that might be toxic or nutrient-deficient for *A. pegreffii*. While FBS has generally been used as the gold standard in cell culture, the ethical concerns of exploiting serum from fetal calves, its refractoriness to complete characterisation, inter-batch variability and reports of contaminants have prompted the development of alternatives, trusted to lead towards FBS successive reduction or exclusion from the routine [26]. FBS has also been recognised as limiting for establishing in vitro culture of other helminths due to its tendency to form precipitates in the media and inter-batch variability that resulted in inconsistent growth-stimulating properties and toxicity [27, 28]. Among six sera tested herein, the fastest growth and the longest culture was observed in chicken and, to some extent, porcine serum combined with *Drosophila* media. While the former enabled robust larval growth, the latter showed inconsistent results for adult maturation time-wise, that is, adulthood was reached at a different time in each repeated experiment. High chicken serum concentration (50%) induced L2 growth incongruent with cuticle shedding, consequently causing L2 mortality, also observed in *Contracaecum multipapillatum* s.l. [29]. In the case of the latter, the authors related the successful exsheathment to higher culture temperature (25 °C), but this is likely not supported by ecological conditions in the case of *Anisakis* spp., since such temperature is above the average for the Mediterranean where *A. pegreffii* is common. Therefore, the concentration of 10 and 20% chicken serum should be used for adult and L2 cultures, respectively, or at least unless a protocol for chemical exsheathment of L2 is devised.

*Anisakis pegreffii* egg development and hatching cascade aligns with that described in soil-transmitted helminths, although the cues for the process initiations remain elusive [30]. Despite previous reports that L3 is the stage in anisakids [31–33] and *H. aduncum* [34] that emerges from the egg, all hatched larvae observed here clearly showed two cuticle sheaths, one tightly enveloping the larvae and the second loosely enwrapping the larvae, whose exsheathment started immediately post-hatching. The L1 cuticle remained within the egg after the hatching of L2, but this is not always clearly discernible. It is important to highlight that Køie et al. [32] observed that live larvae, forced out of eggs in seawater by coverslip pressure shortly before spontaneous hatching, were surrounded by the thin cuticle of the L1. In contrast, infective larvae from naturally hatched eggs were loosely ensheathed in the thick cuticle of the L2. Based on this, Køie et al. [33] concluded that the larva that emerges from the egg is L3 rather than the L2, as previously observed [35]. Køie et al. [33] observed a loose cuticle sheet of the L2, implying that the tight one fitting the larval body is L3 cuticle, and consequently designated the larva as L3. This hypothesis has been retained in successive studies, such as the one describing *A. simplex* hatching [36], where similarly only a single ensheathed cuticle was observed. In contrast, two ensheathed and one cuticle on the larval body were discernible in *Ascaris lumbricoides* L3 hatching from the egg [37]. Adroher et al. [24] and Vales-Vega et al. [29] concluded that L3 of *H. aduncum* and *Contracaecum multipapillatum* s.l. also hatched from eggs, based on a strong similarity of the hatched stage to the infective L3, namely, the presence of the excretory cell, oesophagus, ventriculus and intestine. However, Vales-Vega et al. [29] reported that the excretory gland cell in *C. multipapillatum* s.l. was visible only after approximately 25 days in culture. Using confocal and conventional microscopy, in the present work a distinctively larger cell situated in the proximal second third of the larval body was observed already 72 h post-hatching, but it is speculative whether the cell indeed develops into the excretory gland cell without the use of cell-specific markers. Overall, our observation supports that of Smith and Wootten [35], who emphasised that “the free-living larva which emerges from the egg is apparently a second stage and ensheathed in the cast cuticle of the first moult which evidently takes place within the egg”.

The morphological traits of *A. pegreffii* observed by SEM are typical of those reported in other anisakids [38–41]. Noteworthy is the discharge from the excretory pore throughout all ontogeny stages studied herein (L3, L4, adult), suggestive of the active role of the excretory gland cell. Scattered discharge shows a pattern of the larger clusters of extracellular vesicles (EV) settling at the proximal rim of the pore and smaller EV clusters being expelled more distally. Some individual vesicles were observed on the cuticle of the anterior end, ranging from 50 to 210 nm. The release of L3 *A. pegreffii* EV in culture media has been evidenced by nanoparticle tracking analyses, and the inventory of packed L3 miRNA suggested their role during infections, engaging with cellular proliferation and/or differentiation during the shift from an innate to adaptive immune response, apoptosis and inflammation [42]. While we observed clusters of EVs forming agglomerates of different sizes, the size of individual vesicles coincided with that reported by Cavallero et al. [42]; namely, the authors reported *A. pegreffii* mean EV size of 140.5 ± 0.08 nm (size range 50–350 nm), with two less abundant fractions of larger size (between approximately 210 and 250 nm). While techniques employed herein cannot go further than estimating the overall presence of discharged EV, they confirmed the main role of the excretory gland cell in EV production. In contrast, Boysen et al. [43] observed EVs labelled by a fluorescent lipid analogue (DOPE-Rho) mostly in the *A. pegreffii* buccal cavity and to a lesser degree on the body surface, while it remained inconclusive whether the excretory pore had been involved.

The ultrastructure of *A. pegreffii* uterus and oviduct segments conforms to general tissue appearance in ascarids and related nematodes [44–46]. However, earlier ultrastructural studies, especially those on nematodes, relied on chemical fixation of samples, causing tissue deformities, and tools yielding lower-resolution images that complementarily obscured more inconspicuous details. *Anisakis pegreffii* oocytes abundantly secrete lipid/proteinaceous material in the oviduct lumen during migration towards the uterus, and depletion of material is discernible by the lighter electron density of the cytoplasm in older oocytes, similar to the observation in *Toxocara canis* [47]. Conspicuous fibrillar material enveloped in the Golgi membrane also seems to become more depleted by the time oocytes reach the uterus. We can speculate whether these structures are refractile bodies that have been suggested to form the third cytoplasmic membrane of the zygote eggshell [48], also referred to as the ascaroside layer due to its first description in *A. lumbricoides* [49]. However, while lipid-like granules with traces of proteinaceous material are discernible in early and late oocytes, the granules that could carry vitellus are not. In contrast, the intensive secretion of a vitellus-like substance probably designated for incorporation in mature oocytes is present only in uterine epithelial cells. Since we did not obtain mature oocytes and zygotes in the ultrathin sections, it is not clear at what stage vitellus becomes their constituent. The particular uterine microvillosity and interepithelial contacts that suggest intensive secretion to provide an optimal environment for eggs have not been described previously, warranting further study.

## Conclusions

While the closing of the *A. pegreffii* life-cycle from L3 to reproducing adults is important from many research perspectives (vaccine and drug target research, transgenesis, pathogenesis), further effort in terms of culture media composition and adequate temperature and pH is necessary to optimise the efficient moulting of L2 to infective L3. This study provides valuable new elements of nematode morphology and ultrastructure useful for comparative physiological and evolutionary studies.

## Data Availability

The datasets generated during and/or analysed during the current study are available from the corresponding author on reasonable request.

## Abbreviations

DAPI: 4′,6-Diamidino-2-phenylindole
DMEM: Dulbecco’s modified Eagle medium
EV: Extracellular vesicle
FBS: Foetal bovine serum
L2/L3/L4: Second/third/fourth larval stage
PBS: Phosphate-buffered saline
PCR: Polymerase chain reaction
PS: Penicillin/streptomycin
RFLP: Restriction fragment length polymorphism
SEM: Scanning electron microscopy
SSTNE: Spermidine/spermine/tris/sodium chloride/EDTA (ethylenediaminetetraacetic acid)/EGTA (egtazic acid) lysis buffer
TEM: Transmission electron microscopy

## References

[1] Bušelić I, Trumbić Ž, Hrabar J, Vrbatović A, Bočina I, Mladineo I (2018). Molecular and cellular response to experimental *Anisakis pegreffii* (Nematoda, Anisakidae) third-stage larval infection in rats. Front Immunol.

[2] Hrabar J, Trumbić Ž, Bočina I, Bušelić I, Vrbatović A, Mladineo I (2019). Interplay between proinflammatory cytokines, miRNA, and tissue lesions in *Anisakis*-infected Sprague-Dawley rats. PLoS Negl Trop Dis.

[3] Mladineo I, Hrabar J, Smodlaka H, Palmer L, Sakamaki K, Keklikoglou K (2019). Functional ultrastructure of the excretory gland cell in zoonotic anisakids (Anisakidae, Nematoda). Cells.

[4] Stryiński R, Carrera M, Łopieńska-Biernat E (2019). Tissue-specific proteome of *Anisakis simplex* L4 larvae reveal potential molecular mechanisms involved in parasite development and pathogenicity. Ann Parasitol.

[5] Trumbić Ž, Hrabar J, Palevich N, Carbone V, Mladineo I (2021). Molecular and evolutionary basis for survival, its failure, and virulence factors of the zoonotic nematode *Anisakis pegreffii*. Genomics.

[6] Cavallero S, Bellini I, Pizzarelli A, D’Amelio S (2022). What do *in vitro* and *in vivo* models tell us about anisakiasis? New tools still to be explored. Pathogens.

[7] Stryiński R, Mateos J, Carrera M, Jastrzębski JP, Bogacka I, Łopieńska-Biernat E. Tandem Mass Tagging (TMT) reveals tissue-specific proteome of L4 larvae of *Anisakis simplex* s. s.: Enzymes of energy and/or carbohydrate metabolism as potential drug targets in anisakiasis. Int J Mol Sci. 2022;23:4336. 10.3390/ijms23084336 PMC9027741 35457153

[8] Robertson L, Arcos SC, Ciordia S, Carballeda-Sanguiao N, Mena MDC, Sánchez-Alonso I (2020). Immunoreactive proteins in the esophageal gland cells of *Anisakis simplex* sensu stricto detected by maldi-tof/tof analysis. Genes (Basel).

[9] Palomba M, Paoletti M, Colantoni A, Rughetti A, Nascetti G, Mattiucci S (2019). Gene expression profiles of antigenic proteins of third stage larvae of the zoonotic nematode *Anisakis pegreffii* in response to temperature conditions. Parasite.

[10] Grabda J (1809). Studies on the life cycle and morphogenesis of *Anisakis simplex* (Rudolphi, 1809) (Nematoda: Anisakidae) cultured in vitro. Acta Ichthyol Piscat.

[11] Banning P van. Some notes on a successful rearing of the herring-worm *Anisakis marina* L. (Nematoda: Heterocheilidae). J du Cons /Cons Perm Int pour l’Exploration la Mer. 1971;34:84–8.

[12] Grabda J (1982). Studies on survival and development *in vitro* of *Anisakis simplex* stage 3 larvae in time. Acta Ichthyol Piscat.

[13] Iglesias L, Valero A, Benítez R, Adroher FJ (2001). *In vitro* cultivation of *Anisakis simplex*: Pepsin increases survival and moulting from fourth larval to adult stage. Parasitology.

[14] Wickham H (2016). ggplot2: Elegant Graphics for Data Analysis.

[15] Bartie KL, Taslima K, Bekaert M, Wehner S, Syaifudin M, Taggart JB, et al. Species composition in the *Molobicus* hybrid tilapia strain. Aquaculture. 2020;526:735433.

[16] Nadler SA, Hudspeth DSS (2000). Phylogeny of the Ascaridoidea (Nematoda: Ascaridida) based on three genes and morphology: Hypotheses of structural and sequence evolution. J Parasitol.

[17] Kumar S, Stecher G, Li M, Knyaz C, Tamura K (2018). MEGA X: Molecular evolutionary genetics analysis across computing platforms. Mol Biol Evol.

[18] Altschul SF, Gish W, Miller W, Myers EW, Lipman DJ (1990). Basic local alignment search tool. J Mol Biol.

[19] Bowles J, McManus DP (1993). Rapid discrimination of *Echinococcus species* and strains using a polymerase. Mol Biochem Parasitol.

[20] Bowles J, Blair D, McManus DP. A molecular phylogeny of the human schistosomes. Mol. Phylogenet. Evol. 1995. p. 103–9. 10.1006/mpev.1995.1011 7663756

[21] D’Amelio S, Mathiopoulos KD, Santos CP, Pugachev ON, Webb SC, Picanço M (2000). Genetic markers in ribosomal DNA for the identification of members of the genus *Anisakis* (Nematoda: Ascaridoidea) defined by polymerase-chain-reaction-based restriction fragment length polymorphism. Int J Parasitol.

[22] Holland P (2011). Ecdysozoa: insect and meantodes.

[23] Barker G, Rees H (1990). Ecdysteroids in menatodes. Parasitol Today.

[24] Adroher FJ, Malagón D, Valero A, Benítez R (2004). *In vitro* development of the fish parasite *Hysterothylacium aduncum* from the third larval stage recovered from a host to the third larval stage hatched from the egg. Dis Aquat Organ.

[25] Adroher-Auroux FJ, Benítez-Rodríguez R. Chapter 21 *Hysterothylacium aduncum*. In: Sitjà-Bobadilla A, Bron JE, Wiegertjes GF, Piazzon MC, editors. Fish Parasites A Handb Protoc their Isol Cult Transm. Lings, Great Easton, UK: 5M Book; 2021. p. 311–29.

[26] Piletz JE, Drivon J, Eisenga J, Buck W, Yen S, Mclin M (2018). Human cells grown with or without substitutes for fetal bovine serum. Cell Med.

[27] Leland SE. Studies on the in vitro growth of parasitic nematodes. I. Complete or partial parasitic development of some gastrointestinal nematodes of sheep and cattle. J Parasitol. 1963;49:600–11. 14050236

[28] Smyth JD, Davies Z (1974). In vitro culture of the strobilar stage of *Echinococcus granulosus* (sheep strain): a review of basic problems and results. Int J Parasitol.

[29] Valles-Vega I, Molina-Fernández D, Benítez R, Hernández-Trujillo S, Adroher FJ. Early development and life cycle of *Contracaecum multipapillatum* s.l. from a brown pelican *Pelecanus occidentalis* in the Gulf of California, Mexico. Dis Aquat Organ. 2017;125:167–78. 10.3354/dao03147 28792415

[30] Mkandawire TT, Grencis RK, Berriman M, Duque-Correa MA (2022). Hatching of parasitic nematode eggs: a crucial step determining infection. Trends Parasitol.

[31] Berry GN, Cannon LRG (1981). The life history of *Sulcascaris sulcata* (Nematoda: Ascaridoidea), a parasite of marine molluscs and turtles. Int J Parasitol.

[32] Køie M, Fagerholm H-P (1993). Third-stage larvae emerge from eggs of *Contracaecum osculatum* (Nematoda, Anisakidae). J Parasitol.

[33] Køie M, Berland B, Burt MD (1995). Development to third-stage larvae occurs in the eggs of *Anisakis simplex* and *Pseudoterranova decipiens* (Nematoda, Ascaridoidea, Anisakidae). Can J Fish Aquat Sci.

[34] Køie M (1993). Aspects of the life cycle and morphology of *Hysterothylacium aduncum* (Rudolphi, 1802) (Nematoda, Ascaridoidea, Anisakidae). Can J Zool.

[35] Smith JW, Wootten R (1978). *Anisakis* and anisakiasis. Adv Parasitol.

[36] Højgaard DP (1998). Impact of temperature, salinity and light on hatching of eggs of *Anisakis simlex* (Nematoda, Anisakidae) isolated by a new method, and some remarks on survival of larvae. Sarsia.

[37] Geenen PL, Bresciani J, Boes J, Pedersen A, Eriksen L, Fagerholm H-P (1999). The morphogenesis of *Ascaris suum* to the infective third-stage larvae within the egg. J Parasitol.

[38] Abollo E, Pascual S (2002). SEM study of *Anisakis brevispiculata* Dollfus, 1966 and *Pseudoterranova ceticola* (Deardoff and Overstreet, 1981) (Nematoda: Anisakidae), parasites of the pygmy sperm whale *Kogia breviceps*. Sci Mar.

[39] Quiazon KMA, Santos MD, Yoshinaga T (2013). *Anisakis* species (Nematoda: Anisakidae) of dwarf sperm whale *Kogia sima* (Owen, 1866) stranded off the Pacific coast of southern Philippine archipelago. Vet Parasitol.

[40] Di Azevedo MIN, Knoff M, Carvalho VL, Mello WN, Lopes Torres EJ, Gomes DC (2015). Morphological and genetic identification of *Anisakis paggiae* (Nematoda: Anisakidae) in dwarf sperm whale *Kogia sima* from Brazilian waters. Dis Aquat Organ.

[41] Molina-Fernández D, Adroher FJ, Benítez R (2018). A scanning electron microscopy study of *Anisakis physeteris* molecularly identified: from third stage larvae from fish to fourth stage larvae obtained in vitro. Parasitol Res.

[42] Cavallero S, Bellini I, Pizzarelli A, Arcà B, D’amelio S (2022). A miRNAs catalogue from third-stage larvae and extracellular vesicles of *Anisakis pegreffii* provides new clues for host-parasite interplay. Sci Rep.

[43] Boysen AT, Whitehead B, Stensballe A, Carnerup A, Nylander T, Nejsum P (2020). Fluorescent labeling of helminth extracellular vesicles using an in vivo whole organism approach. Biomedicines.

[44] Foor EW (1968). Zygote formation in *Ascaris lumbricoides*. J Cell Biol.

[45] Wu YJ, Foor EW (1983). Ultrastructure and function of oviduct-uterine junction in *Ascaris suum* (Nematoda). J Parasitol.

[46] Brunanská M (1997). *Toxocara canis* (Nematoda: Ascaridae): the fine structure of the oviduct, oviduct-uterine junction and uterus. Folia Parasitol (Praha).

[47] Brunanská M (1994). *Toxocara canis* (Nematoda, Ascarididae): ultrastructure of the rachis and the ovarian wall. Folia Parasitol (Praha).

[48] Foor EW (1967). Ultrastructural aspects of oocyte development and shell formation in *Ascaris lumbricoides*. J Parasitol.

[49] Lee DL, Leštan P (1971). Oogenesis and egg shell formation in. J Zool.

